# Aerobic glycolysis is important for zebrafish larval wound closure and tail regeneration

**DOI:** 10.1111/wrr.13050

**Published:** 2022-10-05

**Authors:** Claire A. Scott, Tom J. Carney, Enrique Amaya

**Affiliations:** ^1^ Division of Cell Matrix Biology & Regenerative Medicine, School of Biological Sciences, Faculty of Biology, Medicine and Health University of Manchester Manchester UK; ^2^ Institute of Molecular and Cell Biology (IMCB) A*STAR (Agency for Science, Technology and Research) Singapore Singapore; ^3^ Lee Kong Chian School of Medicine, Experimental Medicine Building, Yunnan Garden Campus Nanyang Technological University Singapore Singapore

**Keywords:** appendage regeneration, laconic, lactate, metabolism, Warburg effect, wound healing

## Abstract

The underlying mechanisms of appendage regeneration remain largely unknown and uncovering these mechanisms in capable organisms has far‐reaching implications for potential treatments in humans. Recent studies implicate a requirement for metabolic reprogramming reminiscent of the Warburg effect during successful appendage and organ regeneration. As changes are thus predicted to be highly dynamic, methods permitting direct, real‐time visualisation of metabolites at the tissue and organismal level would offer a significant advance in defining the influence of metabolism on regeneration and healing. We sought to examine whether glycolytic activity was altered during larval fin regeneration, utilising the genetically encoded biosensor, Laconic, enabling the spatiotemporal assessment of lactate levels in living zebrafish. We present evidence for a rapid increase in lactate levels within min following injury, with a role of aerobic glycolysis in actomyosin contraction and wound closure. We also find a second wave of lactate production, associated with overall larval tail regeneration. Chemical inhibition of glycolysis attenuates both the contraction of the wound and regrowth of tissue following tail amputation, suggesting aerobic glycolysis is necessary at two distinct stages of regeneration.

## 1 INTRODUCTION

While some organisms have the ability to heal scarlessly and regenerate fully functional tissues as adults, others possess this ability only in the early developmental stages. Understanding the underlying cellular and molecular processes responsible for successful regeneration may provide essential clues for the development of novel clinical therapies that will promote a better healing and regenerative outcome in humans.

Accumulating evidence indicates metabolism influences complex tissue and cellular processes, including cell differentiation and cell behaviour, and an interest in the role of cell metabolism in regeneration is undergoing a revival, driven largely by the development of new techniques that facilitate addressing the link between metabolism and tissue repair and regeneration. Metabolites, such as lactate, have been reported to act as second messengers in cell signalling,^1^ and a switch from oxidative phosphorylation (OXPHOS) to glycolysis is involved in epithelial to mesenchymal transitions (EMTs), which are important for blastema formation in gecko limb regeneration^2^ and in cancer metastasis.^3^, ^4^ Work in *C. elegans* has shown that reduction of mitochondrial activity has positive effects on ageing,^5^ and multiple studies have linked a switch to glycolytic metabolism to the proliferative potential of stem cells.^6^, ^7^, ^8^ Metabolism further plays an important part in cell identity and differentiation in a variety of settings, including immune cells and neurons.^9^, ^10^ Thus, metabolism plays a wider role in physiology than simply energy production. Given that EMT, proliferation, and differentiation are all processes important for regeneration and wound healing, investigating the potential roles for metabolic reprogramming during regeneration and how these are regulated, may provide insight into how cellular metabolism could be hijacked to facilitate regeneration in humans.

The Warburg effect describes the phenomenon of aerobic glycolysis, in which cells preferentially up‐regulate processing of glucose through the conventionally anaerobic pathway of glycolysis and fermentation while decreasing their mitochondrial activity, regardless of the lower energy yield and availability of oxygen.^11^ This strategy was originally discovered in cancer cells, but has since been implicated in multiple highly proliferative systems, putatively allowing glycolytic and pentose phosphate pathway (PPP) intermediates to support macromolecule synthesis for new cells.^12^ Since regeneration is highly dependent on cell proliferation and growth, one might expect regenerating cells to employ the Warburg effect to provide for the requirements of forming the new tissues of the regenerate. This appears to be the case in multiple regeneration models. The gene profiles of regenerating *Xenopus* tails and adult zebrafish hearts show an up‐regulation of glycolytic genes and a corresponding down‐regulation of mitochondrial genes.^13^, ^14^ This switch to glycolysis has been linked to cell proliferation during cardiomyocyte regeneration.^14^

While varied transcriptomic analyses have suggested that metabolic reprogramming plays a critical role during tissue and appendage regeneration, there is a critical need for improved methods that will facilitate the direct assessment of Warburg‐like metabolism during regeneration with temporal and spatial resolution. Recent developments in genetically encoded sensors for various metabolites have advanced the field of metabolic research and have great potential for exploitation in the zebrafish, due to its impressive regenerative capacity,^15^, ^16^ combined with the transparency of the embryos. Here we aimed to test the potential of a genetic ratiometric Förster resonance energy transfer (FRET)‐based genetic sensor, named Laconic, which is responsive to varying lactate levels.^17^ Given that rising lactate levels can be used as a measure of aerobic glycolysis/Warburg‐like metabolism, we aimed to determine whether this sensor could be used to assess metabolic reprogramming during two models of zebrafish larval fin regeneration, namely after fin fold and tail amputations. We also aimed to ask whether altering metabolic reprogramming, using chemical inhibitors targeting glycolysis or lactate dehydrogenase, affected the speed or efficiency of wound closure and/or fin and tail regeneration.

## 2 MATERIALS & METHODS

### 2.1 Cloning

Laconic/pcDNA3.1(−) was a gift from Luis Felipe Barros (Addgene plasmid #44238; http://n2t.net/addgene:44238; RRID:Addgene_44238).^17^ The Laconic genetic sensor in Laconic/pcDNA3.1(−) was cloned into the pCS2+ vector and the p3 vector from the pTransgenesis system,^18^ using standard restriction digest and sticky end recombination methods. In some cases, complimentary primers were designed and annealed to produce short sticky end fragments that were inserted into constructs in order to generate additional complementary restriction sites. For specific restriction enzymes, inserts, and buffers used, see Table S1.

For the transgene cassettes, the modular cloning system pTransgenesis^18^ based on the Gateway system of cloning^19^ was used, and recombination was facilitated with the Gateway LR Clonase II Enzyme Mix (Invitrogen, 11791) according to manufacturer instructions: incubated overnight with the LR clonase enzyme at 23°C, followed by inactivation by addition of 0.5 μl Proteinase K at 37°C for 10 min. 3 μl of the reaction was transformed into 30–50 μl chemically competent DH5α E. coli cells (Invitrogen) as detailed previously.

### 2.2 Zebrafish husbandry

Adult AB strain wild‐type and *Tg[ubb:laconic]* ^*lkc1*^ zebrafish (*Danio rerio*) were maintained at 28°C with a 14 h light/10 h dark cycle. Embryos collected from in‐crosses were staged as described in Kimmel *et al*.^20^ All animal experiments were performed in compliance with NACLAR Guidelines of Singapore overseen by the Biological Resource Centre of A*STAR (IACUC Protocol Number 140924), and Home Office guidelines UK. In all cases, embryos were raised in 1X E3 embryo medium as described in Cold Spring Harbour Protocols, or 1X egg water consisting of 60 μg/mL sea salts (Sigma Aldrich S9883), supplemented with 0.1% Methylene Blue unless stated otherwise.

### 2.3 mRNA microinjections

Wild‐type strain AB zebrafish embryos were injected at the one‐cell stage into the cell cytoplasm with 1 ng laconic sensor mRNA in nuclease‐free water with phenol red. Laconic sensor mRNA was synthesised from pCS2 plasmids linearised with NotI (NEB), with mMESSAGE mMACHINE SP6 Transcription Kit (Ambion) and purified with lithium chloride (LiCl) extraction.

### 2.4 Generation of transgenic lines

Wild‐type strain AB zebrafish embryos were injected at the one‐cell stage into the cell cytoplasm with 25pg tol2 mRNA and 25 pg circular plasmid in 1 nL. Tol2 mRNA was synthesised from pT3‐Tol2 linearised with SmaI (NEB) with mMESSAGE mMACHINE T3 Transcription Kit (Ambion) and purified with LiCl extraction. Injected embryos with the strongest expression of mosaic GFP were grown into adults and out‐crossed to screen for germline transmission.

### 2.5 Biochemical lactate assay

A commercially available colorimetric lactate assay kit (MAK064, Sigma Aldrich) was used and protocol adapted for embryonic samples. Lactate in the sample reacts with the enzyme mix provided in the kit, the product of which interacts with the supplied lactate probe to produce colour (A_570_) and fluorescence (excitation/emission = 535/587 nm). We chose to identify lactate concentration by measuring the colorimetric product of the enzymatic reaction with lactate at an absorbance of 570 nm.

Samples were prepared by flash freezing on dry ice and macerating 25 dechorionated eggs or embryos with a plastic micropestle in 45 μl 2:2:1 acetonitrile:methanol:dH_2_O at −20°C or pre‐chilled on dry ice. Samples were then centrifuged at 4°C at 15000rcf for 10 min, the supernatant collected into a new tube and stored at −20°C until use in the assay. 5 μl of the embryo supernatant was used per reaction.

A standard curve was set up using known concentrations of a lactate standard (0, 2, 4, 6, 8 and 10 nM per reaction) with the addition of 5 μl 2:2:1 to each reaction in order to control for any background or change in enzyme activity caused by the buffer.

Triplicate reactions were set up otherwise according to manufacturer instructions, with a minimum of three biological repeats. Reaction incubation time was extended to 3 h, and absorbance at 570 nm (A_570_) was read on a microplate reader (BioTek Synergy H1) in triplicate to give a total of nine readings per sample.

### 2.6 Fin fold and tail amputations

2 days post fertilisation (dpf) embryos were mounted in 1% low melting agarose (Invitrogen 16520100) supplemented with 0.04% MS‐222 (tricaine, Sigma Aldrich E10521) on a glass microscope slide for imaging with an upright microscope or in a 35 mm glass‐bottomed dish (Thermo Scientific Nunc) for imaging with an inverted microscope, and imaged pre‐amputation. Amputations were made while embryos were mounted in agarose with either a size 10 or 15 scalpel blade, the agarose surrounding the fins excavated, and the embryos covered with media. Fin fold amputations were performed just distal to the tip of the notochord, and tail amputations were oriented using the pigment gap and transected just distal to the circulatory loop of the caudal vein. Images were then taken at various timepoints post amputation with the embryos being de‐mounted from the agarose and kept in 1X egg water or 1X E3 at 28°C between imaging timepoints of longer than one hour.

For experiments where imaging immediately post amputation was not required, 2dpf embryos were not mounted in agarose, and instead amputated in a droplet of 1X egg water supplemented with 0.04% MS‐222 on a glass microscope slide, transferred to 1X egg water with or without drug treatment within 5 min of amputation, and maintained at 28°C.

### 2.7 Microscope sample preparation

Embryos were visually screened using a fluorescent dissecting microscope for transgenic expression and dechorionated manually with forceps. Embryos were mounted in 1% low melting agarose (Invitrogen 16520100) supplemented with 0.04% MS‐222 (tricaine, Sigma Aldrich E10521) on a microscope slide for imaging with an upright microscope, or in a 35 mm glass‐bottomed dish (Thermo Scientific Nunc) for imaging with an inverted microscope.

### 2.8 Microscopy

Images were acquired on an AxioImager.M2 upright microscope (Zeiss) for Figures 3, 4, 6 and 7 using a 5X/0.16 EC Plan‐Neofluar, 10X/0.3 EC Plan‐Neofluar, or 20X/0.4 Corr LD Plan‐Neofluar objective was used as specified. Zeiss filter sets for CFP (BS455) and FITC/mCherry (DBS525/50 + 650/100) were utilised for Laconic imaging, with Violet (430 nm) excitation from a Colibri 7 LED fluorescent light source. Imaging software: Zen Blue 2.3 Pro. The images were collected using a 2.8 Megapixels (Axiocam 503) colour camera at 14‐bit on the black‐and‐white setting at room temperature.

Laconic imaging for Figure 8 was acquired on an Eclipse Ti inverted microscope (Nikon) with a 4X/0.13 Plan Fluor PhL DL objective using a SpetraX light engine (Lumencore) with individual Semrock emission filters for eCFP (480/30) and eYFP (535/30), and excitation with Blue (440/20) LED fluorescent light source and filter. The images were collected using a Retiga R6 (Q‐Imaging) CCD camera at 14‐bit. Imaging software: NIS Elements AR.46.00.0. Mechanised point visiting was used to allow multiple positions to be imaged and environment was maintained at 28°C.

Images for immunohistochemistry and phalloidin staining were acquired on an LSM800 (Zeiss) upright confocal microscope using a 20X/0.5 N‐Achroplan WD (water) objective. Emission was collected at 400‐550 nm with excitation laser 488 nm for pNM‐488, 561‐700 nm emission with 561 nm excitation for rhodamine phalloidin, and 400‐454 nm emission with 405 nm excitation for DAPI. Imaging software: Zen Blue 2.3 Pro. The images were collected using two‐channel multi‐alkali PMT detectors at 8‐bit, pinhole 1 AU, with Z‐stacks of 2.44 μm slice intervals.

### 2.9 Image analysis

All processing of images for calculating ratio and measuring fluorescence or ratio was conducted in Fiji (version 2.0.0). Average background was subtracted and threshold applied to remove the remaining background, then Laconic ratio was calculated by dividing the 428 nm emission channel by the 485 nm emission channel using the Image Calculator function. Pseudo‐colouring was applied using Lookup Table “16 colors”.

Quantification of fin width or regrowth length for amputation experiments was conducted with Fiji. Regrowth length was defined as the perpendicular distance from the tip of the notochord to the distal fin fold edge.

### 2.10 Pharmacological treatment

2dpf embryos were maintained in 0.5X E2 medium (half strength modification of the E2 embryo medium described in Cunliffe [2003]) in place of 1X E3 embryo medium. Drug treatments were maintained until one‐hour post amputation.

Antimycin A (AA, Sigma Aldrich A8674) was dissolved to make a stock solution of 5 mM in dimethyl sulfoxide (DMSO, Sigma Aldrich D8418) and diluted 1:1000 in E2 medium for a working concentration of 5 μM with a final concentration of 0.1% DMSO.

A stock concentration of 500 mM Sodium oxamate (Sigma Aldrich O2751) was dissolved in distilled water fresh for each use and diluted in E2 supplemented with 0.04% MS‐222 (tricaine, Sigma Aldrich E10521) to a working concentration of 10, 150 or 200 mM. For amputation experiments examining the initial hour of regeneration, embryos were amputated in media containing oxamate at a concentration of 150 or 200 mM; for those regarding the whole of the regeneration process, embryos were placed into media containing 10 mM oxamate immediately following amputation and kept in the drug until assessment at 120 hpa.

A stock concentration of 4 mM GNE‐140 (Sigma Aldrich SML2580) was dissolved in DMSO and diluted in E2 supplemented with 0.04% MS‐222 (tricaine, Sigma Aldrich E10521) to a working concentration of 40 and 400 mM. For amputation experiments examining the initial hour of regeneration, embryos were amputated in media containing GNE‐140 at a concentration of 400 mM; for those regarding the whole of the regeneration process, embryos were placed into media containing 40 mM GNE‐140 immediately following amputation and kept in the drug until assessment at 120hpa.

For sodium azide (NaN_3_, Sigma Aldrich S2002) treatment, powder form of the drug was dissolved in 1X phosphate buffered saline (PBS, Sigma Aldrich P5493) fresh for each use at a stock concentration of 1.5 M and diluted in E2 supplemented with 0.04% MS‐222 to a working concentration of 15 mM or 25 mM.

2‐Deoxy‐D‐glucose (2DG, Sigma Aldrich D8375) was dissolved in distilled water to a stock solution of 250 mM and diluted 1:10 in 1X egg water with methylene blue to produce a working concentration of 25 mM. In amputation experiments, embryos were placed into media containing 2DG immediately following amputation and 2DG treatment was maintained from 0 hpa until 72 hpa (Figure S1), then washed out and the embryos placed in new 1X egg water with methylene blue, as longer treatment results in embryo mortality.

For Laconic imaging experiments of the first‐hour post amputation, amputations were made while embryos were mounted in agarose with either a size 10 or 15 scalpel blade, then agarose surrounding the fins was excavated and the embryos covered with the treatment solution. Images were then taken 10 min post amputation. For experiments over the whole of regeneration, embryos were amputated in a droplet of tricaine solution on a glass microscope slide and transferred to inhibitor treatment within five minutes post amputation. For immunohistochemistry samples, 2dpf embryos were amputated in a droplet of the oxamate and tricaine solution on a glass microscope slide with a size 10 or 15 scalpel blade, incubated for 10 min at room temperature, then fixed as described below.

### 2.11 Immunohistochemistry

10 AB strain wild‐type embryos per condition per experiment were fixed in either 4% paraformaldehyde (PFA, Sigma Aldrich F8775) in 1X phosphate buffered saline (PBS, Sigma Aldrich P5493) at room temperature for 2 h or 95% methanol (MeOH, Sigma Aldrich 34,860)/5% glacial acetic acid (GAA, Sigma Aldrich A6283) at −20°C for 4 h.

In brief: if fixed in 95% MeOH/5% GAA washes were done with PBDT (1XPBS/1%BSA/2%DMSO [dimethyl sulfoxide, Sigma Aldrich D8418]/0.5%Tween, if fixed in 4% PFA washes were in PBST (1XPBS/0.1%Tween or Triton). Samples were systematically rehydrated in methanol washes, then washed in PBDT or PBST as specified by the fixation method, followed by acetone cracking at −20°C for 7 (PFA fix) or 14 (MeOH/GAA fix) minutes. Blocking was in 2% donkey serum for 2 h at room temperature. Primary antibody, rabbit α‐phosphomyosin light chain II (Ser19),^21^, ^22^ Cell Signalling Technology #3671), was added to fresh block at a dilution of 1:250 and incubated overnight at 4°C. Secondary antibody, donkey α‐rabbit Alexa Fluor 488 (Invitrogen R37118), was added to fresh block at a dilution of 1:500 and incubated overnight at 4°C. DAPI (Invitrogen D1306) was added 1:1000 (10 nM) during the first half an hour of wash post‐secondary antibody addition. Samples were transferred to 50% glycerol (Sigma Aldrich G5516) in 1X PBS and stored at 4°C.

Phalloidin staining: rhodamine phalloidin (Invitrogen R415) was made up as 40X stock solution in methanol and added in a 1:40 dilution to 4% PFA fixed samples, either alone directly after fixation or in tandem with secondary antibody addition.

### 2.12 Statistical analysis

GraphPad Prism 8 was used for statistical testing, with sample numbers exceeding 6 in all experiments, and each experiment was replicated three or more times. Column or grouped statistics and analyses of differences between means were implemented for all data sets. For column statistics, two‐tailed unpaired t‐tests with assumed Gaussian distribution were used. Two‐way ANOVA was used with Sidak's multiple comparisons test to compare means between groups. All data are presented as mean ± s.d., and differences were considered significant to * at *P* < 0.05, ** at *P* < 0.01, *** at *P* < 0.001, and **** at *P* < 0.0001. Not significant (ns) was considered *P* ≥ 0.05, 95% confidence interval.

## 3 RESULTS

### 3.1 Laconic can be used to monitor lactate levels in zebrafish embryos and larvae

**FIGURE 1:**
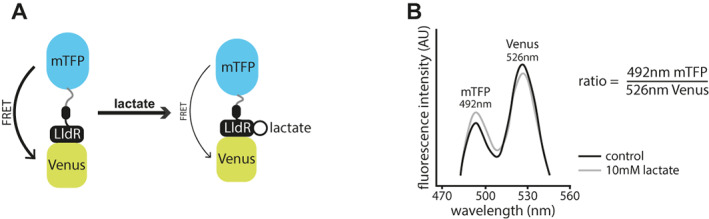
Schematic of the Laconic genetically encoded sensor for lactate. (A) Schematic depiction of the Laconic genetically encoded FRET‐based sensor. A conformational change upon lactate binding to the LldR lactate binding region fused to the Venus chromophore induces a change in energy transfer efficiency from mTFP to Venus (adapted from^17^ Figure 1B). (B) Graph depicting emission spectra of Laconic. When bound to lactate, fluorescence intensity detected in the range of mTFP increases and that of Venus decreases due to reduced FRET efficiency, thereby increasing the Laconic ratio as calculated by mTFP/Venus (adapted from^17^).

The genetically encoded biosensor, Laconic, was originally developed and tested on cells in culture^17^ and has since been used in mouse brains,^23^ but has not been used in whole organisms, such as the zebrafish. The FRET‐based sensor is composed of a lactate binding region, the bacterial transcription factor LldR, linked with the fluorescent proteins mTFP and Venus.^17^ Upon binding of lactate, a conformational change decreases the FRET efficiency of energy transfer from the donor chromophore, mTFP, to the acceptor chromophore, Venus (Figure 1A). By exciting mTFP and measuring the emission from both mTFP and Venus, one can form a ratio to depict the changes in lactate with temporal and spatial resolution. The mTFP/Venus ratio (the Laconic ratio) increases with lactate levels (Figure 1B).

We devised a positive control, whereby we treated embryos 17 h post fertilisation (hpf) with antimycin A (AA), a mitochondrial OXPHOS inhibitor, which acts to drive glucose into aerobic glycolysis and thus conversion into lactate. We first established the action of AA was indeed increasing lactate levels by measuring the concentration of lactate in treated embryos using a commercial biochemical assay kit. When comparing lactate concentrations in embryos before and after 10 min of treatment with either AA or DMSO, we found that AA‐treated samples gave significantly higher readings of lactate concentrations (Figure S1). From this, we were confident that AA treatment would provide a satisfactory method to test the efficacy of the Laconic sensor.

In order to confirm Laconic reports lactate dynamics in the zebrafish embryo, we injected *in vitro* transcribed *laconic* mRNA into one‐cell stage zebrafish embryos and imaged them before treatment and after one hour of treatment, either with AA or DMSO vehicle control. The Laconic ratio increased significantly in response to AA but not DMSO (Figure S1). Thus, we concluded that Laconic can successfully report lactate levels in zebrafish embryos.

We then generated a transgenic line (*Tg[ubb:laconic]* ^*lkc1*^) expressing Laconic under the control of the *ubiquitin B* promotor (*ubb*)^24^ in order to visualise lactate levels over the course of larval fin regeneration. This Laconic transgenic line showed a higher Laconic FRET ratio following treatment with AA, indicating it reliably reports lactate levels in embryos and larvae (Figure S2). As further confirmation, we performed a second positive control using an alternate mitochondrial inhibitor, sodium azide (NaN_3_), which acts on complex IV of the electron transport chain,^25^ similarly blocking OXPHOS, and driving the cell toward glycolysis. As with AA, one hour of treatment with NaN_3_ increased the Laconic ratio significantly compared to PBS control treatment. Furthermore, as NaN_3_ is a reversible inhibitor, we also measured Laconic ratio after 24 h of recovery following washout of the drug and observed lactate levels had returned to those of controls (Figure S2).

### 3.2 Lactate levels increase transiently immediately post‐larval fin fold amputation

**FIGURE 2:**
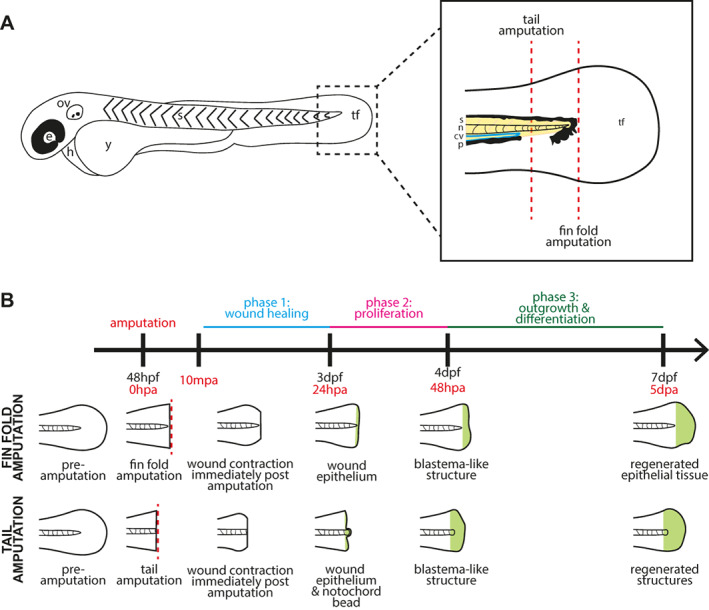
Schematic diagrams of the fin and tail amputation models of zebrafish embryo regeneration. (A) Schematic drawing of a two‐day post fertilisation zebrafish embryo. e: eye, ov: otic vesicle, h: heart, y: yolk, s: somites, tf: tail fin. Dashed box indicates the area shown enlarged in the box to the right, which depicts the tail region with amputation planes for fin fold and tail amputations (dashed red lines). A fin fold amputation site is positioned just distal to the tip of the notochord and excision is limited epithelial tissue and mesenchymal cells, while a tail amputation is oriented using the pigment gap and circulatory loop of the caudal vein, severing notochord, neural tube, and muscle in addition to epithelial tissue. s: somites (yellow), n: notochord (pink), cv: caudal vein (blue), p: pigment (black), tf: tail fin fold. (B) General timeline and schematic illustration of phases of regeneration following amputation. Embryos are imaged at various time points depending on the specific experiment between ten minutes post amputation (10mpa) and full regeneration at five days post amputation (5dpa).

We next assessed whether lactate levels change following two types of larval fin injury, namely following distal fin fold amputation, where only distal epidermal fin tissue was excised, and following tail amputations, where many additional tissues are transected, including the notochord and spinal cord (Figure 2A). Both types of injury induce three similar phases of tissue response and regeneration (wound healing, proliferation, and outgrowth and differentiation, Figure 2B) that are mostly comparable to adult fin regeneration, albeit on a much faster time scale.^26^, ^27^, ^28^ Upon amputation, most regenerative responses form a highly proliferative structure termed the blastema.^29^ Since regeneration involves the regrowth of lost tissue, it would stand to reason that larval tail regeneration would be dependent on the generation of significant amounts of new biomass. As such, appendage regeneration is a good candidate for the Warburg effect.^13^ Thus, we aimed to image lactate levels, as a proxy for the Warburg effect, during regeneration following larval zebrafish fin fold and tail amputations utilising the *Tg[ubb:laconic]* ^*lkc1*^ line.

**FIGURE 3:**
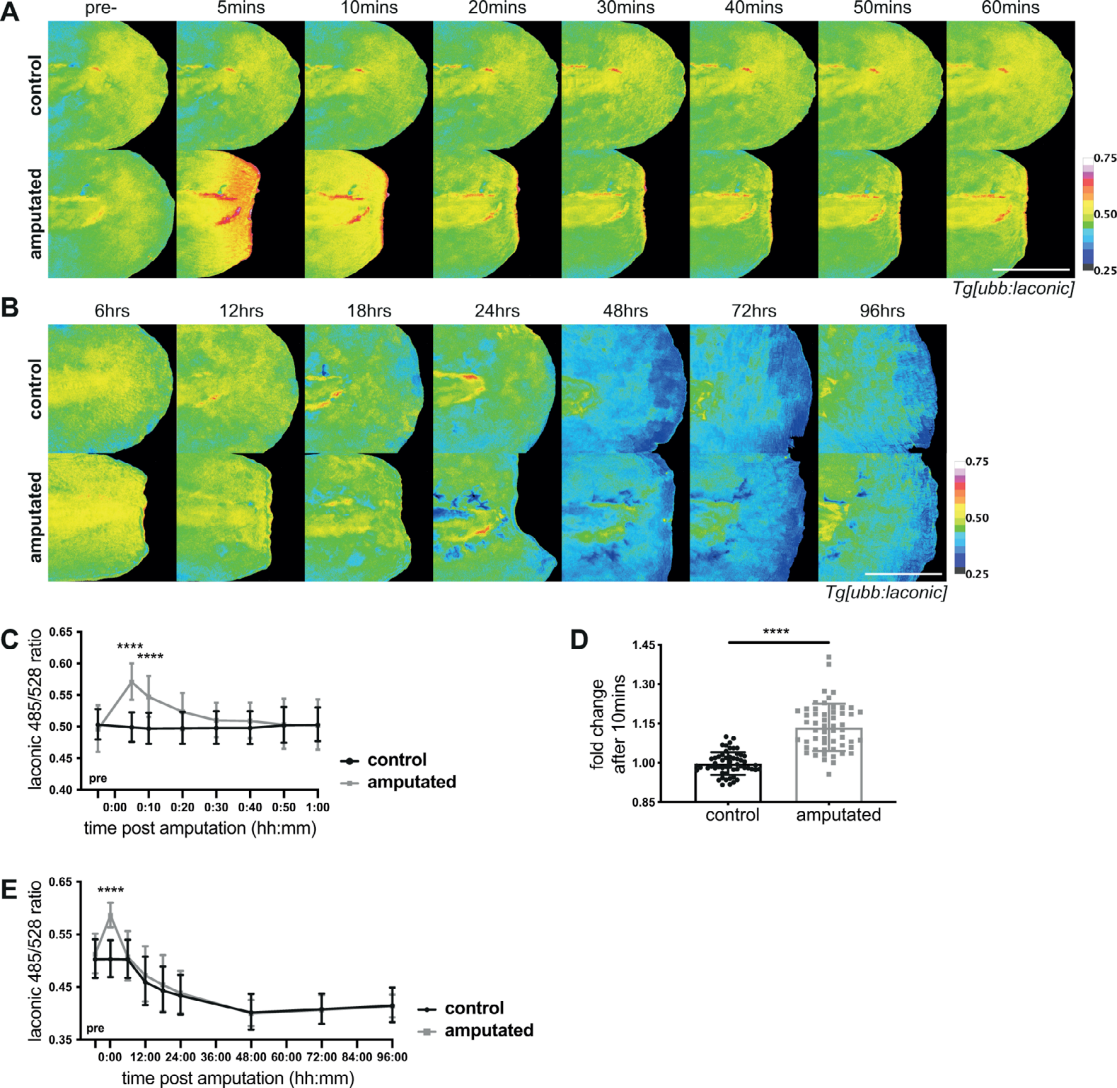
Lactate levels in fin fold regeneration. A) Micrographs of representative *Tg[ubb:laconic]* ^*lkc1*^ embryos tails at 48 hpf imaged pre‐amputation and the same individual embryo followed over the course of one‐hour post amputation, pseudocoloured to show Laconic ratio. (B) Micrographs of representative transgenic *Tg[ubb:laconic]* ^*lkc1*^ embryos tails amputated at 48 hpf and imaged at given time‐points over the course of regeneration until five days post amputation, pseudocoloured to show Laconic ratio. (C) Graph showing quantification of raw Laconic ratios pre‐treatment and over the course of one‐hour post amputation. Two‐way ANOVA to calculate significance, *n* = 18. (D) Graph showing fold change between pre‐amputation ratio and 10 min post amputation. Students' t‐test to calculate significance, n = 56. (E) Graphs showing quantification of raw Laconic ratios pre‐treatment and at various timepoints post amputation. Two‐way ANOVA to calculate significance, *n* = 18. All scale bars represent 200 μm. Differences were considered significant to * *P* < 0.05, ** *P* < 0.01, *** *P* < 0.001, **** *P* < 0.0001, and ns *P* ≥ 0.05.

We found that the Laconic ratio increased immediately following distal fin fold amputation, peaking within the first five minutes post amputation (mpa) and returning to control levels by 50 mpa (Figure 3A,C), with a significant change at 10 mpa when compared to the pre‐amputated value (Figure 3D). Spatially, lactate levels were raised in a broad gradient from the wound border, up to approximately 80 μm into the fin. For the remaining period of distal fin fold regeneration, there was no significant difference between amputated and un‐amputated controls (Figure 3B,E).

### 3.3 Inhibition of lactate production impairs wound contraction following fin fold amputation

Given that the elevated lactate levels we measured are coincident with the rapid wound healing phase following injury, we decided to test whether aerobic glycolysis might play a crucial role during the actomyosin‐driven wound healing phase, which takes place within the first ten minutes after injury^27^ (also see: Supplementary Movie 1). Glycolysis is able to produce ATP more rapidly than OXPHOS, which is why the fastest contracting muscle fibres, which are also actomyosin based, are largely glycolysis‐based in their metabolism.^30^ Potentially this is an explanation for the transient burst of lactate and glycolysis activity after injury, as a strategy for swiftly producing large quantities of ATP to fuel the energy‐intensive contraction of the actomyosin cable during wound closure.

**FIGURE 4:**
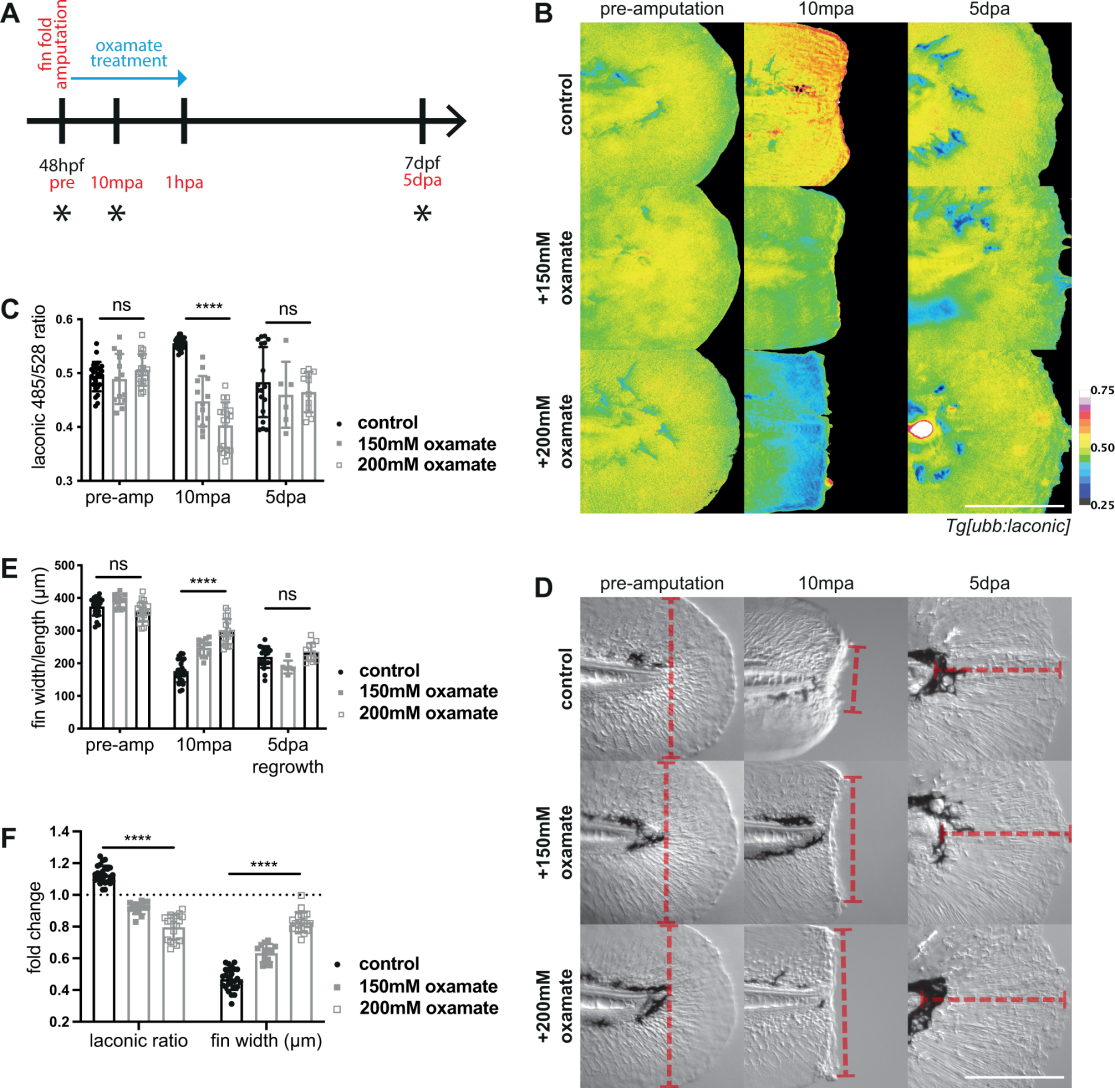
Lactate dehydrogenase inhibition in wound healing. (A) Schematic of the experimental design. Embryos were amputated in the treatment solution and incubated for one hour before washing out the drug, and maintained until regeneration was complete at 120 hpa. Blue arrow indicates period of oxamate treatment and black asterisks indicate time points for imaging. (B) Micrographs of representative *Tg[ubb:laconic]* ^*lkc1*^ embryos tails at 48 hpf imaged pre‐amputation, 10 min post amputation with treatment with oxamate or water control, and five‐days post amputation, pseudocoloured to show Laconic ratio. (C) Graph showing quantification of raw Laconic ratios pre‐, 10 min post‐, and five‐days post‐amputation. Two‐way ANOVA to calculate significance, *n* = 25 (control), *n* = 13 (150 mM oxamate), *n* = 19 (200 mM oxamate). (D) DIC micrographs of representative transgenic *Tg[ubb:laconic]* ^*lkc1*^ embryos tails as in (B) with examples of measurements (red dashed line) taken for fin width and length quantification. Pre‐amputation and wound width taken to be from edge to edge of the fin fold just distal to the notochord along the amputation plane; regrowth taken from the end of the notochord to the most distal edge of the fin fold, perpendicular to amputation plane. (E) Graph showing measured fin widths/length in micrometres pre‐, 10 min post‐, and five‐days post‐amputation. Fin length measured from the tip of the notochord to the distal edge of the fin fold. Two‐way ANOVA to calculate significance, *n* = 25 (control), *n* = 13 (150 mM oxamate), *n* = 19 (200 mM oxamate). (F) Graph showing fold change (10 mpa value divided by pre‐amputation value) of Laconic ratio and fin width in micrometres in the first 10 min of amputation with treatment with oxamate or water control. Dotted line on the Y axis marks a fold change of 1 (no change). Two‐way ANOVA to calculate significance, *n* = 25 (control), *n* = 13 (150 mM oxamate), *n* = 19 (200 mM oxamate). All scale bars represent 200 μm. Differences were considered significant to * *P* < 0.05, ** *P* < 0.01, *** *P* < 0.001, **** *P* < 0.0001, and ns *P* ≥ 0.05.

We tested this hypothesis by inhibiting aerobic glycolysis and assessing the effects on the actomyosin cable contraction at the wound. To do this, we transiently inhibited the activity of lactate dehydrogenase (LDH) using the competitive inhibitor, sodium oxamate,^31^ during the wound healing phase and up to an hour post fin fold amputation, and took images and movies during the wound healing process (Figure 4A). LDH converts pyruvate to lactate and regenerates NAD+, permitting continued glycolysis independent of mitochondrial activity. We reasoned that using chemical inhibitors provides a powerful method of transiently inhibiting LDH activity, not easily afforded by alternative methods, such as standard genetic knockouts or knockdown, which are unlikely to be compatible with survival long‐term. To test the efficacy of the drug, we first asked whether oxamate affected the rapid increase in lactate levels following injury. Oxamate treatment successfully prevented the increase in lactate post‐injury in a dose‐dependent manner (Figure 4B,C). We then asked if LDH inhibition affected wound healing / closure or subsequent fin fold regeneration. Indeed, oxamate treatment potently inhibited/delayed wound contraction following injury (Supplementary Movie 2). To quantify this effect, we measured fin width across the plane of amputation as an assessment of wound contraction and we found that oxamate treatment resulted in the wound remaining significantly wider at 10mpa (Figure 4D,E) and decreasing in width significantly less than controls (Figure 4F). Removing oxamate from the media one‐hour post amputation (hpa) did not affect overall regeneration, and the fins appeared similar to controls in terms of Laconic ratio and fin regrowth length at 5‐days post‐amputation (Figure 4B‐E). These data suggest that rapid glycolysis activity immediately following amputation is required for the rapid contraction of the wound margin within the first 10 min post‐injury, but the embryos are still able to recover following temporary inhibition of LDH and this transient inhibition is not detrimental to long‐term viability of the larvae and regeneration of the distal fin folds (Figure 4E). This reflects our previous Laconic data, which showed only a transient rise in lactate levels following distal fin fold amputation and elevated lactate levels are not sustained during the fin fold regeneration phase. To further confirm our hypothesis that LDH activity and aerobic glycolysis are important for wound contraction, we utilised an alternate LDH inhibitor, GNE‐140,^32^, ^33^ to transiently inhibit LDH activity during the rapid wound healing phase following distal fin fold amputation. We found that GNE‐140 also strongly inhibited the rapid contraction of the wound post amputation, comparable to the inhibition seen following oxamate treatment (Figure S3, Supplementary Movies 3 and 4).

**FIGURE 5:**
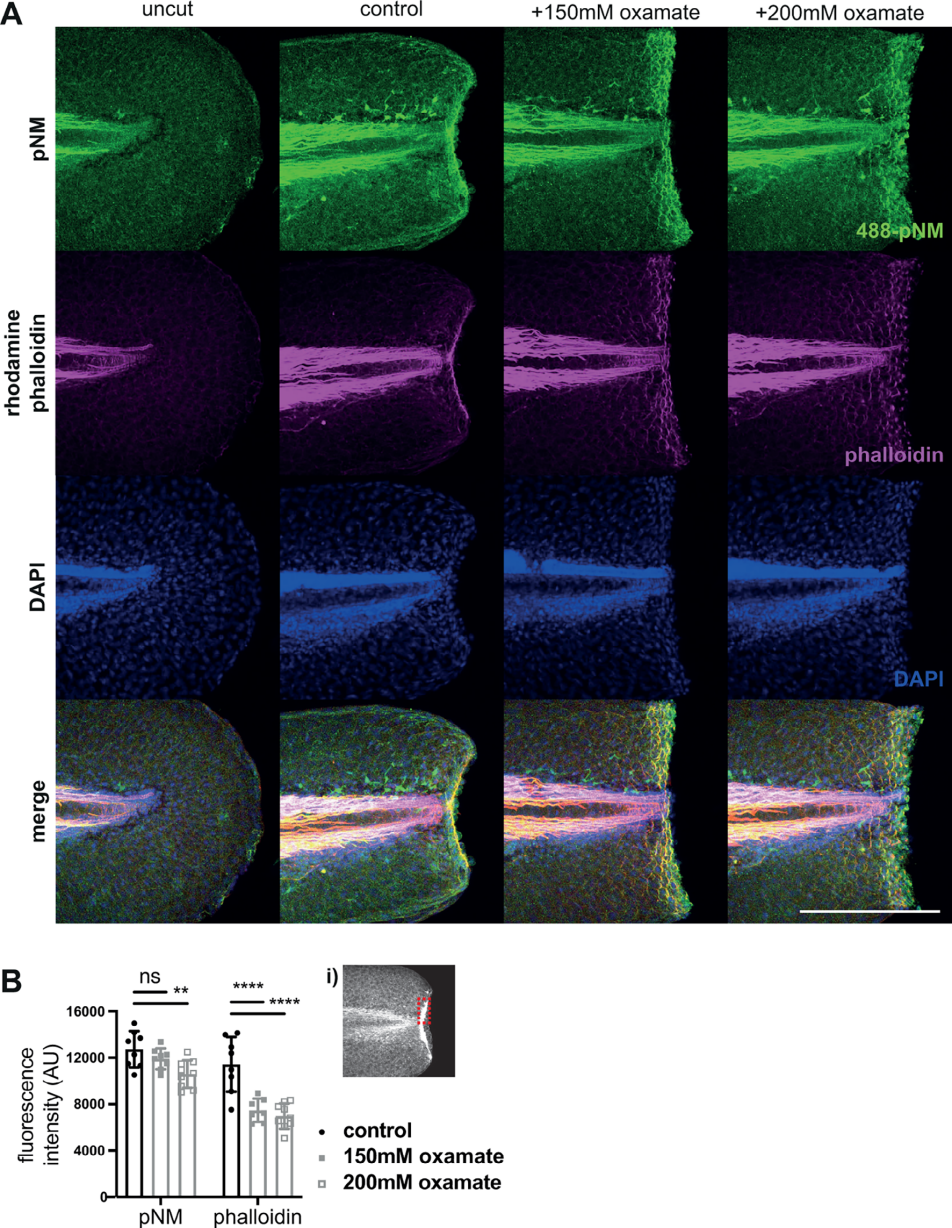
Effect of lactate dehydrogenase inhibition on actomyosin cable contraction in wound healing. (A) Maximal intensity confocal micrograph projections of representative embryos tails at 48 hpf fixed and stained for phospho‐non muscle myosin light chain II (pNM), Actin, DAPI, and merged, at 10 min post amputation. (B) Graphs showing quantification of fluorescence intensity at the wound border of phalloidin Actin staining and immunofluorescent pNM staining. Inset (i) denotes example of region measured. Two‐way ANOVAs used to calculate significance, ** *P* < 0.01, **** *P* < 0.0001, ns *P* ≥ 0.05, *n* = 8. All scale bars represent 200 μm.

To test the importance of rapid glycolysis for actomyosin activity, we stained oxamate‐treated and control amputated embryos for phosphorylated non‐muscle myosin (pNM) and actin using immunohistochemistry and phalloidin, respectively. Phosphorylation of non‐muscle myosin was unaffected by treatment with lower levels of oxamate, and only slightly reduced with higher concentrations (Figure 5A,B). However, actin at the wound border was significantly diminished with both high and low concentrations of oxamate treatment. Myosin phosphorylation requires only a single ATP to donate the phosphate group for each myosin, and therefore is not the most energetically demanding process, whereas active contraction requires one molecule of ATP for each myosin stroke cycle.^34^ We propose that it is this contraction that requires the use of glycolysis. It may be that actin stabilisation and condensation at the site of action requires activity of myosin, and the lack of actin remodelling may indicate a loss of active contraction.

### 3.4 Wound contraction in fin fold amputation is not dependent on oxidative phosphorylation

Our findings suggest that wound contraction is highly reliant on glycolysis, but this does not preclude a similar requirement for OXPHOS during wound contraction. Therefore, we endeavoured to determine whether inhibition of OXPHOS using NaN_3_ similarly affected wound contraction following larval tail fin amputation.

**FIGURE 6:**
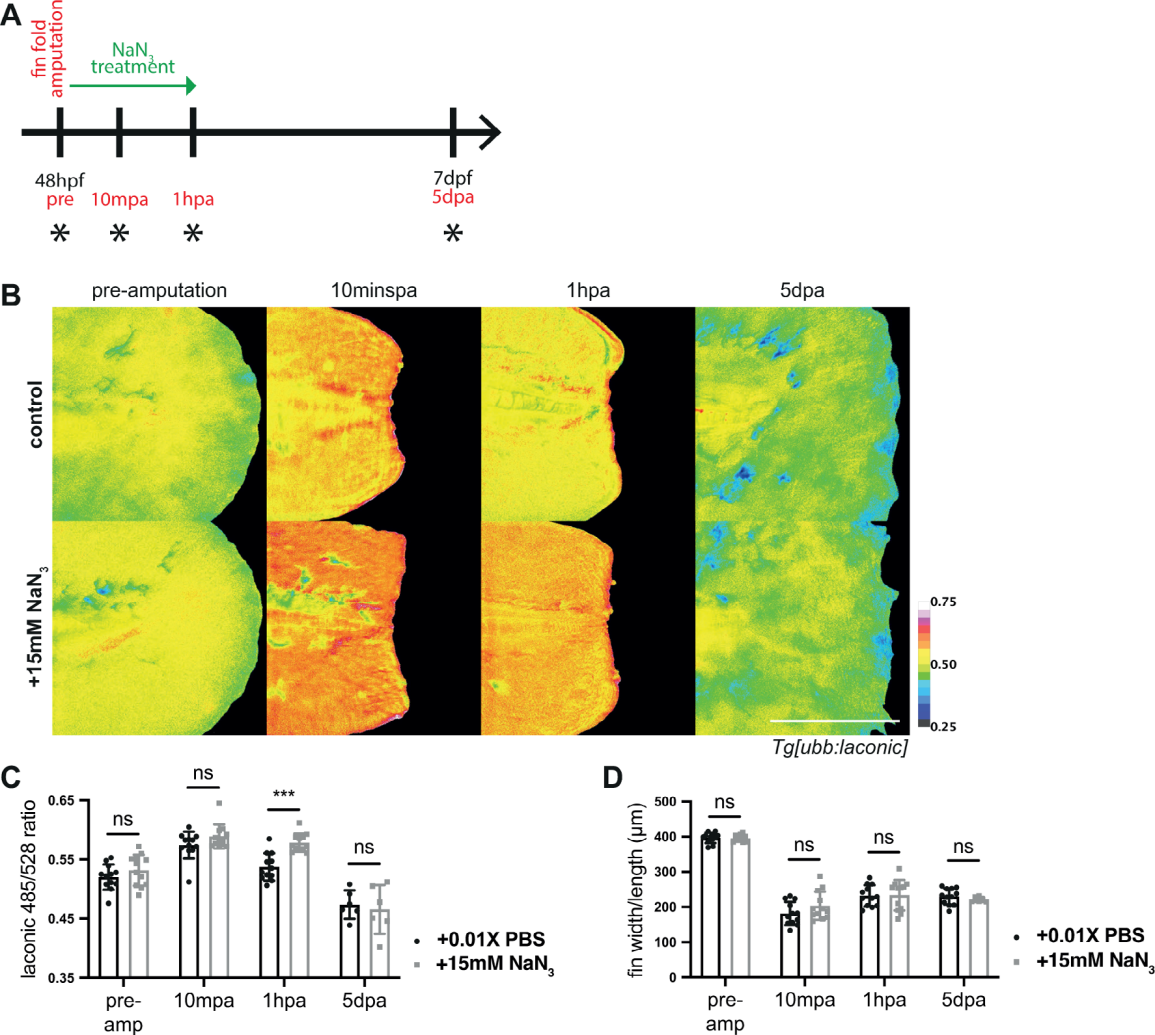
Mitochondrial inhibition in wound healing. (A) Schematic of the experimental design. Embryos were amputated in the treatment solution and incubated for one‐hour before washing out the drug, and maintained until regeneration was complete at 120 hpa. Green arrow indicates period of sodium azide (NaN_3_) treatment and black asterisks indicate time points for imaging. (B) Micrographs of representative *Tg[ubb:laconic]* ^*lkc1*^ embryos tails treated at 48 hpf for one‐hour post amputation with 15 mM NaN_3_, to induce prolonged glycolysis and lactate production, or PBS control. Images were acquired at pre‐amputation, 10 min, one‐hour, and five‐days post amputation, and pseudocoloured to show Laconic ratio. (C) Graph showing quantification of raw Laconic ratios pre‐, 10 min post‐, and five‐days post‐amputation. Two‐way ANOVA to calculate significance, *n* = 12. (D) Graph showing measured fin widths/length in micrometres pre‐, 10 min post‐, and five‐days post‐amputation. Fin length measured from the tip of the notochord to the distal edge of the fin fold. Two‐way ANOVA to calculate significance, *n* = 12. All scale bars represent 200 μm. Differences were considered significant to * *P* < 0.05, ** *P* < 0.01, *** *P* < 0.001, **** *P* < 0.0001, and ns *P* ≥ 0.05.

We performed fin fold amputations on 2dpf *Tg[ubb:laconic]* ^*lkc1*^ embryos and immersed them immediately in NaN_3_ treatment, and then measured Laconic ratios after one‐hour of treatment (Figure 6A). We found no significant difference in treated versus control embryos, although the NaN_3_ embryos trended toward having higher levels of lactate (Figure 6B,C). Additionally, there was no difference in fin widths or wound contraction (Figure 6D), thus we concluded that a reduction of mitochondrial activity does not affect the rapid wound contraction phase following injury.

By 1hpa control embryos have reduced in Laconic ratio, while NaN_3_ treated embryos continued producing elevated lactate levels (Figure 6B,C). After washing out the drug at this point and allowing embryos to regenerate their fin folds, we imaged both conditions and found that there was no difference in Laconic ratio or fin regrowth at five days post amputation (dpa) (Figure 6B‐D). This suggests an overabundance of lactate and a transient loss in mitochondrial OXPHOS activity during the early wound healing phase has no consequence on either wound closure or overall regeneration, while a reduction in lactate and glycolysis activity negatively impacts the rapid wound healing phase. Moreover, the apparent lack of requirement for OXPHOS in wound contraction suggests that this process is predominantly dependent on glycolysis.

### 3.5 Lactate is elevated in the notochord bead following tail amputations

**FIGURE 7:**
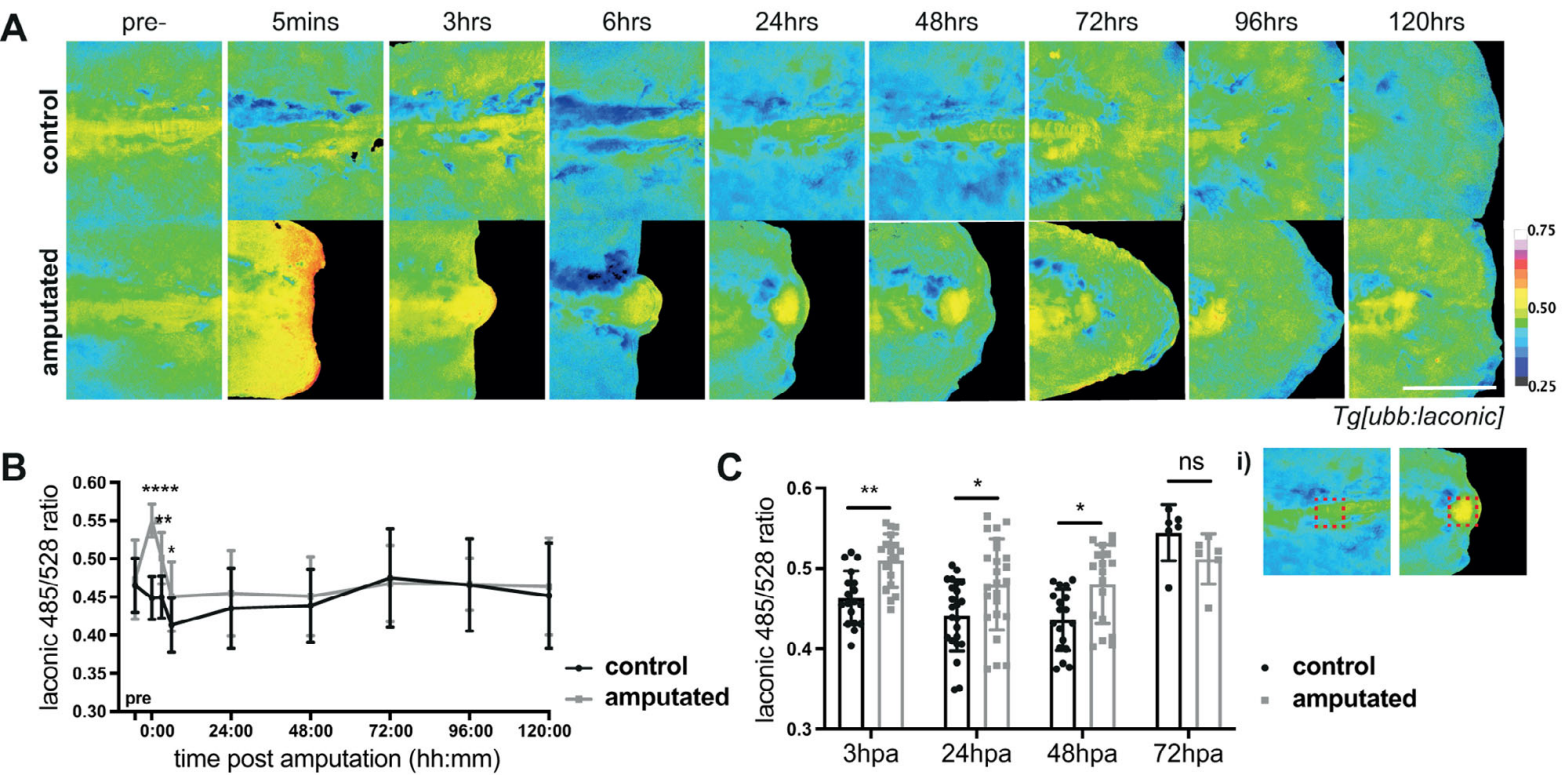
Lactate levels in tail regeneration. (A) Micrographs of representative *Tg[ubb:laconic]* ^*lkc1*^ embryos tails at 48 hpf imaged pre‐amputation and at various time‐points over the course of regeneration (note that images show representative embryos rather than following an individual embryo), pseudocoloured to show Laconic ratio. (B) Graphs showing quantification of raw Laconic ratios pre‐treatment and at various timepoints post amputation. Two‐way ANOVA to calculate significance, *n* = 24. (C) Graph showing quantification of raw Laconic ratios specifically in the notochord bead region (as indicated in (i)). Students' t‐test to calculate significance, *n* = 24. Scale bar represents 200 μm. Differences were considered significant to * *P* < 0.05, ** *P* < 0.01, *** *P* < 0.001, **** *P* < 0.0001, and ns *P* ≥ 0.05.

After seeing that lactate levels rise dramatically, but only transiently, during the wound healing phase following fin fold amputation, we asked whether there was any evidence for metabolic reprogramming during the later regeneration phase following tail amputation (Figure 2B). During both larval fin fold and tail regeneration, a proliferative second phase occurs; however, tail amputation additionally involves the formation of a “notochord bead”, which arises from extruding notochord sheath cells and displays high rates of proliferation,^27^ which could be linked to changes in metabolism. Overall, early lactate dynamics were similar following both fin fold and tail amputations, with a near instant and rapid increase in Laconic ratio (Figure 7A,B). However, the initial elevated lactate persisted following tail amputations (24 h post amputation (hpa)) when compared to distal fin fold amputations. In particular, we found sustained higher Laconic ratios in the notochord bead until 48 hpa (Figure 7C). This blastema‐like structure begins formation at 12 h post amputation and blastema genetic markers begin to be lost after 48 hpa^25^, ^27^; therefore, higher lactate levels correlated with the presence of the proliferative notochord bead and blastema‐like structure.

### 3.6 Inhibition of glycolysis prevents successful tail regeneration, but not fin fold regeneration

An up‐regulation in glycolytic enzymes with an accompanying reduction in mitochondrial activity occurs in proliferating cardiomyocytes of regenerating zebrafish hearts^14^ and genes involved in glycolysis and the PPP are significantly up‐regulated during *Xenopus* tadpole tail regeneration.^13^ Along with the elevation of aerobic glycolysis in the tail notochord bead noted previously, this prompted us to assess the potential role for aerobic glycolysis during zebrafish larval tail regeneration versus fin fold regeneration.

**FIGURE 8:**
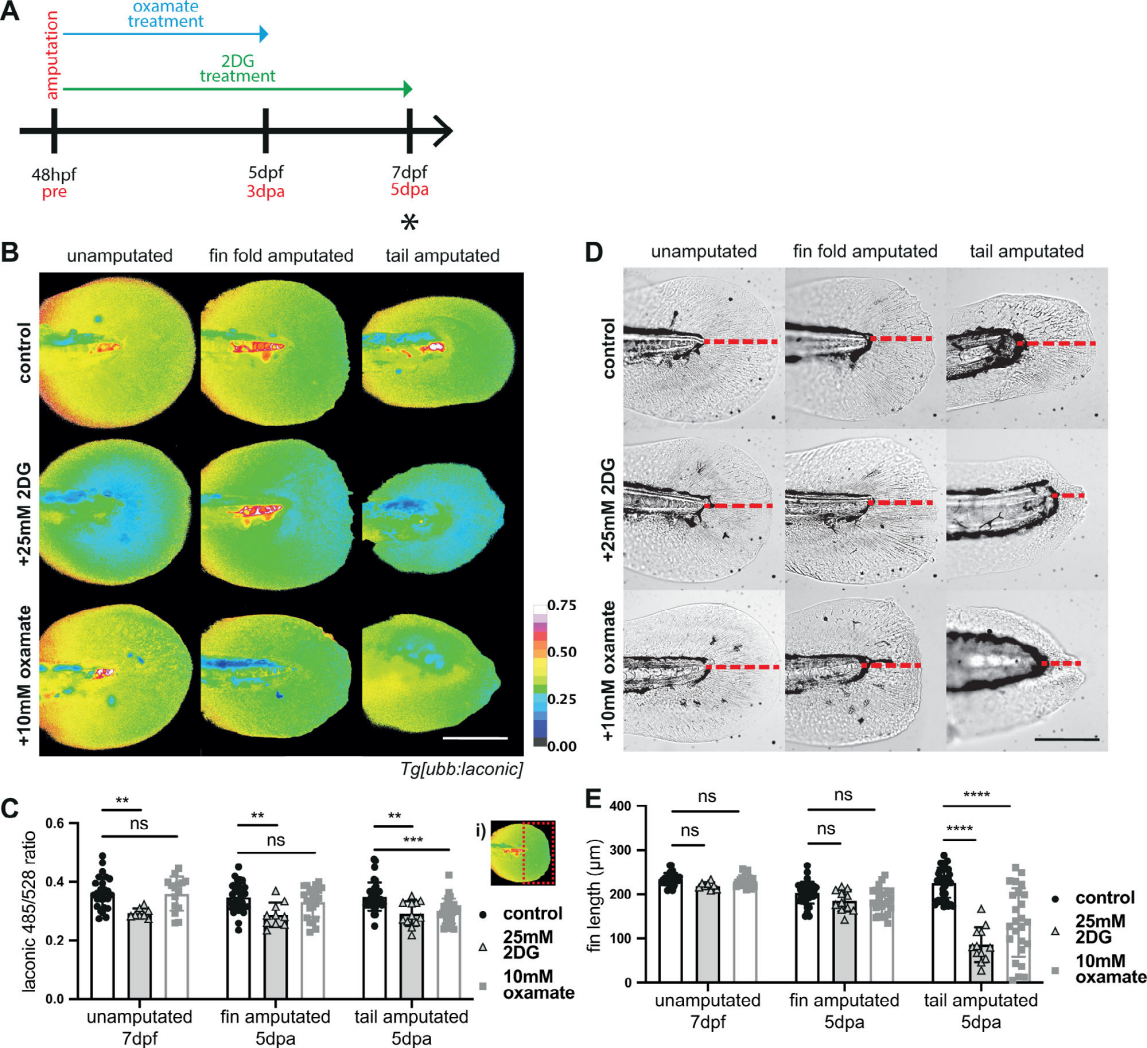
Glycolysis inhibition in regeneration. (A) Schematic of the experimental design. Embryos were amputated and incubated in treatment solution for 72 h or 120 h as indicated by the blue (oxamate) and green (2DG) arrows. Black asterisks indicates time point for imaging, at 5 dpa upon completion of regeneration. (B) Micrographs of representative *Tg[ubb:laconic]* ^*lkc1*^ larvae tails, pseudocoloured to show Laconic ratio. Imaged at 7 dpf or 120 hpa when amputated at 48 hpf, treated for the first 72 h post amputation with 25 mM 2DG, for the full 120 h of regeneration with 10 mM oxamate, or with a control. (C) Graph showing quantification of raw Laconic ratios at 7 dpf or 120 hpa when amputated at 48 hpf and treated for the first 72 h post amputation with 25 mM 2DG, 10 mM oxamate, or control. Two‐way ANOVA to calculate significance, *n* = 13. White dashed box in inset (i) shows example of area measured for quantification of Laconic ratio in (C). (D) Brightfield images of representative *Tg[ubb:laconic]* ^*lkc1*^ larvae tails at 7 dpf or 5 dpa when amputated at 48 hpf, treated for the first 72 h post amputation with 25 mM 2DG, for the full 120 h of regeneration with 10 mM oxamate, or with a control. Red dashed line indicates measurement taken for fin length. (E) Graph showing fin length measurements taken at 7 dpf or 120 hpa when amputated at 48 hpf and treated for the first 72 h post amputation with 25 mM 2DG or with a vehicle control. Two‐way ANOVA to calculate significance, *n* = 13. All scale bars represent 200 μm. Differences were considered significant to * *P* < 0.05, ** *P* < 0.01, *** *P* < 0.001, **** *P* < 0.0001, and ns *P* ≥ 0.05.

We thus treated 2 dpf amputated *Tg[ubb:laconic]* ^*lkc1*^ embryos over the course of regeneration with 2DG, a competitive inhibitor of hexokinase and glucose‐6‐phosphate isomerase, two critical enzymes in the glycolytic pathway^35^ and analysed the resulting tail lengths at 5 dpa (Figure 8A). To confirm the efficacy of 2DG on lactate production, we measured lactate levels at 120 hpa after treatment for the first 72 hpa and verified that 2DG caused a significant decrease in Laconic ratio, and therefore lactate level, in both distal fin fold and tail amputation conditions at 120 hpa and in 7 dpf unamputated controls (Figure 8B,C). Given that 2DG can potently reduce lactate levels in both our regeneration assays, we asked if regeneration of either the fin fold or tail was compromised in its presence. Tail amputated embryos did not regenerate when treated with 2DG (Figure 8D), resulting in a significantly shorter tail length at 120 hpa (Figure 8E). In contrast, distal fin fold amputations, though averaging a shorter length of regrowth than controls, were not significantly affected (Figure 8D,E), consistent with the lack of lactate increase in this post‐wound healing phase. Later elevated Laconic ratios and therefore lactate levels were seen only in the notochord bead of tail amputations (Figure 7A,C), while fin fold amputations lacked the formation of this structure and showed no significant difference in Laconic ratio throughout the duration of the regeneration phase (Figure 3E). Thus, the importance of glycolysis is likely related to the formation of the blastema‐like notochord bead structure. There was no difference between 2DG treated and control unamputated embryo fin lengths (Figure 8C,D), suggesting glycolysis is not essential for normal embryo fin development.

To support the findings with 2DG, we additionally utilised oxamate, the LDH inhibitor we used previously to attenuate the initial burst of lactate production occurring during wound healing. The longer length of the regeneration experiments, compared to the wound healing assay, required a lower concentration of oxamate (10 mM), as the higher concentrations used previously (150‐200 mM) were not compatible with survival over multiple days of exposure. Indeed, we noted that even at 10 mM oxamate treatment visibly affected the swimming ability of the larvae as early as 24 h after treatment. Nevertheless, we noted significantly reduced lactate levels in the tail amputated larvae treated with 10 mM oxamate treatment at 120 hpa, although this reduction was not seen in fin fold amputated or unamputated larvae (Figure 8B,C). As with the experiments with 2DG, oxamate treatment over the entirety of regeneration attenuated regrowth following tail amputation, while allowing distal fin fold amputated larvae to reach lengths comparable to those of the amputated controls (Figure 8D,E). Likewise, oxamate‐treated unamputated embryos showed no difference in fin lengths when compared to controls (Figure 8D,E), supporting the conclusion that rapid or aerobic glycolysis is not critical for normal fin formation of the embryos.

Finally, to support the findings that attenuating LDH activity affected tail regeneration, but not distal fin regeneration, we also treated embryos with the alternative LDH inhibitor, GNE‐140. As with oxamate, a lower concentration of the inhibitor was required (40 μM) to sustain viability over the five days of treatment. Also like oxamate, GNE‐140 treatment significantly reduced the regenerative ability of the larvae following tail amputation (Figure S4). Again, though in general regenerative length was shorter than controls, GNE‐140 did not significantly affect regeneration following distal fin fold amputation (Figure S4).

Thus, we hypothesise that aerobic glycolysis plays an essential role during larval tail regeneration, which involves the regeneration of many tissues, including the spinal cord and notochord, but aerobic glycolysis is not similarly required for distal fin fold regeneration, which is not associated with long‐term elevation of lactate levels and is not dependent on a blastema‐like structure.

## 4 DISCUSSION

### 4.1 The future of genetically encoded sensors in vivo

We have shown the genetically encoded sensor, Laconic, can successfully report lactate levels in zebrafish larvae. Use of genetic sensors of metabolites in vivo allows the assessment of the dynamic changes of metabolism with spatial resolution on individual animals, a feat that is impossible to emulate using biochemical methods. It further permits imaging with more refined temporal resolution, such as with time‐lapse movies, and expressing the sensor under tissue‐specific promoters additionally enables selective interrogation of metabolites in distinct subtypes of cells during complex multicellular processes, such as organogenesis or regeneration.

Whilst effective, the Laconic sensor suffered from low fluorescence intensity in our transgenic line, which limited its sensitivity. There may also be additional interference from autofluorescence, especially in cells with high pigment or yolk content, and the multicellular nature of organisms, which additionally affects the sensitivity of the sensor. We were able to confirm the ability of Laconic to report lactate levels with a biochemical lactate assay, and, in the future, combining genetically encoded metabolite sensors with biochemical assays, whole embryo metabolomics and/or MALDI mass spectrometry imaging will produce a complementary array of data, with the sensors providing a broader depiction of the temporal and spatial changes in metabolism while metabolomic approaches supplying a more comprehensive dataset of information of a large range of metabolites at given timepoints.

Other in vivo studies have also demonstrated the applicability of genetically encoded biosensors for measuring metabolite dynamics in zebrafish. The iNap1 sensor was used to show that NADPH levels decrease following embryonic fin amputation and co‐localises with hydrogen peroxide (H₂O₂). This was interpreted as a result of dual oxidase (DUOX) activity, which consumes NADPH while generating H₂O₂.^36^ Activity of the pentose PPP, specifically the enzyme glucose‐6‐phosphate dehydrogenase, is the main contributor to NADPH production,^37^ and thus iNap sensors could in future studies be also utilised as an indicator of the Warburg effect alongside Laconic.

### 4.2 A role for aerobic glycolysis in early wound healing and formation of a blastema‐like structure

In both distal larval fin fold and tail amputations the rapid increase in lactate levels within minutes following amputation occurs prior to the proliferative phase of regeneration, and correlates with the actomyosin contraction of the wound margin. Our chemical inhibitor experiments suggest that this rapid rise in lactate levels is necessary for wound contraction. More specifically, inhibition of LDH results in failure of the wound to contract and an attenuation of actin re‐organisation and concentration at the wound border through purse‐string action of myosin on actin. This reduction was not seen upon inhibition of mitochondrial OXPHOS activity, suggesting this process is primarily dependent on glycolysis. However, we did not directly measure OXPHOS activity nor did we measure the speed of wound closure at timepoints prior to 10 mpa, thus we are unable to rule out the possibility that OXPHOS inhibition does not result in a brief acceleration or delay in wound contraction prior to 10mpa.

One might ask whether this rapid rise in lactate levels immediately after amputation is the result of aerobic glycolysis or anaerobic glycolysis? Two lines of evidence point toward aerobic glycolysis. The first is that, as mentioned above, inhibition of OXPHOS has little effect on wound closure, thus rapid oxygen consumption due to OXPHOS is unlikely to be occurring. The second is that, based on evidence in *Xenopus* tadpoles, where there is a rapid rise in oxygen levels at the wound immediately following tadpole tail amputations,^38^ it is unlikely that the wound margin in our zebrafish embryos is becoming anoxic or hypoxic immediately after injury. However, given we did not specifically assess oxygen levels, we cannot conclude this with certainty.

A similar role for aerobic glycolysis has been shown in the brains of mice, whereby upon neuronal excitation, glycolysis temporarily exceeds the rate of oxidative metabolism needed to provide for the rapid increase in energy demand.^39^ Furthermore, the process of enucleation in erythrocytes requires contraction of an actomyosin ring and is prevented when aerobic glycolysis is blocked by inhibition of the glycolytic enzymes glyceraldehyde 3‐phosphate dehydrogenase (GAPDH) or LDH.^40^ Thus, we propose that aerobic glycolysis provides a means of rapid ATP production necessary for driving the energy consuming process of actomyosin‐mediated wound contraction minutes after amputation, showcasing the growing importance of considering the impact of metabolism on the regulation of biomechanics. Further elucidation of this hypothesis would benefit from the use of another biosensor to measure ATP levels, such as PercevalHR,^41^ to confirm whether ATP levels drop dramatically specifically at the wound margin when aerobic glycolysis is inhibited during the rapid wound healing phase. An alternative role for lactate may be for stabilisation of actomyosin cable itself, as previous work has shown that sodium lactate is able to stabilise actomyosin in vitro against temperature perturbations.^42^, ^43^ While we have found that both larval fin amputations and tail amputations lead to a rapid metabolic shift toward aerobic glycolysis, which is necessary for the rapid wound healing phase, it remains unknown whether other forms of injury are also associated with a similar shift in metabolism. However, given the conserved involvement of actomyosin / purse‐string mediated contraction during rapid wound healing events in both single cell and multicellular injury models,^44^ we would predict that such a metabolic shift may be a general hallmark of rapid wound healing mechanisms that are energy demanding, but evidence to support this assertion will require further investigation.

During the subsequent regeneration phases following wound healing, aerobic glycolysis, as indicated by localised elevated lactate levels, is once again implicated in the notochord bead/blastema during tail regeneration. In contrast, we saw no significant increase in lactate levels at these later stages during fin fold regeneration. The larval fin fold “blastema” does not play a specific role in proliferation as it does in adult fin regeneration, and instead proliferation occurs in a more spatially distributed manner.^27^ Tail amputation, however, is more akin to a canonical appendage regenerative response, in that multiple tissues must be replenished, including the notochord, spinal cord and skeletal muscle. The blastema‐like structure formed following tail amputations expresses genes typically associated with the blastema in other regenerative organisms and is partly made up of extruded notochord cells, creating the “notochord bead”.^28^ We show that the blastema‐like notochord bead has elevated lactate levels as early as 3 hpa and continues until 48 hpa. The raised lactate levels correlate temporally with the blastema, returning to control levels after 48 hpa as the regenerant enters the third phase of regeneration, characterised by differentiation and progressive scaling back of proliferation.^26^, ^27^ Other work has also shown elevated levels of glycolysis gene expression and decreased mitochondrial activity during zebrafish heart regeneration. Inhibition of glycolysis with 2DG resulted in a reduction of proliferating cardiomyocytes,^14^ indicating this metabolic switch to glycolysis is required for regrowth. Increased expression of glycolysis genes has also been observed in zebrafish following larval tail amputation, with inhibition with 2DG resulting in abnormal blastema formation,^45^ and we additionally find that activity of the glycolytic enzymes hexokinase and LDH are required for larval tail regeneration. Thus, aerobic glycolysis appears to be required for successful regeneration through the formation or output of the blastema‐like notochord bead. As 2DG acts very early in glycolysis, it is able to impact other glucose metabolic pathways, including the pentose PPP and the hexosamine biosynthetic pathway (HBP). Though we demonstrate a reduction of lactate levels with 2DG and oxamate treatment, suggesting the importance of glycolysis specifically during regeneration, it is possible that other pathways may also be involved in zebrafish tail regeneration or that these inhibitors are impacting on pathways unrelated to glycolysis, which then impact on regeneration. Indeed, requirement of the HBP during larval appendage regeneration has been proposed.^45^

The regrowth of the epidermis following fin fold amputations, however, does not require the function of these glycolytic enzymes and achieved regrowth comparable to controls despite glycolytic inhibition. It is unclear at this point if this different reliance on aerobic glycolysis between the two amputation models reflects diversity in the constituent cell types being regenerated or the differing anabolic needs for regeneration of the fin fold, versus overall regeneration of many tissue types. Intriguingly, recent findings suggest that a similar metabolic switch also occurs following adult fin regeneration in zebrafish, and inhibition of this switch results in failure in blastema formation in the adult fin as well.^46^

Thus, our work suggests that aerobic glycolysis is important at two distinct points following injury: the first being within minutes following injury, during the rapid wound healing phase and the second during the tail regeneration phase. Though a blastema is typically highly proliferative, there is an absence of raised lactate levels in any region aside from the notochord bead during the proliferative phase of fin and tail regeneration. Aside from being a product of the Warburg effect, lactate may also have a direct effect on blastema formation and function, such as acting as a second messenger. For example, lactate has recently been shown to mediate magnesium uptake into the mitochondria,^1^ which in turn has been reported to have a stimulatory effect on oxidative metabolism and may affect mitochondrial calcium flux.^47^ The downstream targets and signalling stimulated by lactate in this instance remain unknown, their elucidation a possible direction for future studies. Other future work could also look into whether proliferative cells are reduced in glycolysis‐inhibited tail amputations, as is the case in zebrafish heart regeneration.^14^

The underlying mechanisms governing metabolic reprogramming during tail regeneration remain unknown. Both hypoxia‐inducible factor‐1α (HIF1α) signalling and the embryonic form of pyruvate kinase (PKM2) have been implicated in the switch of induced pluripotent stem cells to glycolytic metabolism, leading to their de‐differentiation.^48^, ^49^ More broadly, there is increasing evidence that hypoxic conditions and reactive oxygen species (ROS) influence glycolytic switching. HIF1α signalling is also sufficient for inducing reprogramming to glycolytic metabolism in mouse embryonic stem cells^50^ and is known to have a positive effect on glycolysis, such as in cancer^51^ and macrophages.^52^ Further, H₂O₂ has been shown to positively regulate glycolysis in cancer cells.^53^ Illuminating the molecular pathways involved in successful regeneration, such as the relationship between H₂O₂ and glycolysis, will assist in determining the logic of metabolic reprogramming in different phases of regeneration. Zebrafish imaging approaches, combining an expanding genetically encoded biosensor toolbox with high regenerative capacity, offer a unique system to determine principles of metabolic programming in regeneration.

AUTHOR CONTRIBUTIONS

Conceptualisation: Claire A. Scott, Tom J. Carney, Enrique Amaya; Methodology: Claire A. Scott; Validation: Claire A. Scott; Formal analysis: Claire A. Scott; Investigation: Claire A. Scott; Writing ‐ original draft: Claire A. Scott; Writing ‐ review & editing: Claire A. Scott, Tom J. Carney, Enrique Amaya; Supervision: Tom J. Carney, Enrique Amaya; Project administration: Enrique Amaya; Funding acquisition: Enrique Amaya.

## FUNDING INFORMATION

This work was supported by a PhD studentship from the A*STAR ARAP PhD programme to C.A.S. and a Medical Research Council Research Project Grant [MR/L007525/1] to E.A.

## CONFLICT OF INTEREST

The authors declare no competing or financial interests.

## Supporting information


**Figure S1** Laconic positive controls as mRNA injections. (A) Graph of lactate levels in 2dpf embryos (calculated using a standard curve) after 10 min of treatment with 0.1% DMSO or 5 μM AA compared to a pre‐treatment baseline level. One‐way ANOVA to calculate significance, *n* = 36. (B) Micrographs of representative ~19hpf wild‐type embryos injected with laconic mRNA at the one‐cell stage before and after 60 min of treatment with 0.1% DMSO or 5 μM AA, pseudocoloured to show Laconic ratio. (C) Graph showing Laconic ratios over time during treatment with 0.1% DMSO or 5 μM AA, normalised to pre‐treatment value. Two‐way ANOVA to calculate significance, *n* = 4. (D) Graph showing quantification of ratio change as fold change (post‐treatment ratio divided by pre‐treatment ratio). Students' t‐test to calculate significance, *n* = 4. All scale bars represent 200 μm. Differences were considered significant to * *P* < 0.05, ** *P* < 0.01, *** *P* < 0.001, **** *P* < 0.0001, and ns *P* ≥ 0.05

**Figure S2** Laconic positive controls as a transgenic line. (A) Micrographs of representative *Tg[ubb:laconic]* ^*lkc1*^ embryos tails at 48hpf before and after treatment with 0.1% DMSO or 5 μM AA, pseudocoloured to show Laconic ratio. (B) Graph showing raw Laconic ratios pre‐treatment and after treatment with 0.1% DMSO or 5 μM AA. Two‐way ANOVA to calculate significance, *n* = 6. (C) Graphs showing quantification of ratio change after 60 min of treatment with 0.1% DMSO or 5 μM AA as fold change (ratio after treatment divided by pre‐treatment value). Students' t‐test to calculate significance, *n* = 6. (D) Micrographs of representative *Tg[ubb:laconic]* ^*lkc1*^ embryos tails at 48hpf before and after 60 min of treatment with 0.01X PBS or 25 mM sodium azide (NaN_3_), and after 24 h recovery post wash out of the drug, pseudocoloured to show Laconic ratio. (E) Graph showing raw Laconic ratios pre‐treatment, after 60 min of treatment with 0.01X PBS or 25 mM NaN_3_, and after 24 h of recovery after drug wash out. Two‐way ANOVA was used to calculate significance, *n* = 8. (F) Graph showing quantification of ratio change after 60 min of treatment with 0.01X PBS or 25 mM NaN_3_ as fold change (ratio after treatment divided by pre‐treatment value). Students' t‐tests were used to calculate significance, *n* = 8. All scale bars represent 200 μm. Differences were considered significant to * *P* < 0.05, ** *P* < 0.01, *** *P* < 0.001, **** *P* < 0.0001, and ns *P* ≥ 0.05.

**Figure S3** Further inhibition of lactate production during fin fold amputation. (A) Brightfield images of 2dpf WT control (no treatment) compared with 200 mM oxamate‐ and 400 μM GNE‐140‐treated embryos at 0minspa and 20minspa. Red dashed lines indicate example measurements taken for quantification. The scale bar represents 200 μm. (B) Graph showing percentage contraction at 10minspa of wound width at 0minspa. One‐way ANOVA to calculate significance.

**Figure S4** Further inhibition of lactate production over the whole of regeneration. (A) Brightfield images of representative WT embryos at 5dpa (7dpf), treated with 0.1% DMSO (control), 10 mM oxamate, or 40 μM GNE‐140. Red dashed line indicates measurements taken for quantification of regrowth. The scale bar represents 200 μm. (B) Graph comparing inhibited (10 mM oxamate or 40 μM GNE‐140 treatment) with control (0.1% DMSO treatment) embryos in the unamputated, fin fold amputated, and tail amputated conditions at 5dpa (7dpf). Two‐way ANOVA to calculate significance.

**Movie 1** Time‐lapse movie generated of the rate of wound healing/contraction following distal fin amputation under control / untreated condition over 12 min. 360 still images were taken every 2 s over 12 min and rendered as a time‐lapse movie using iMovie at 40 frames per second, giving a 9‐s time‐lapse movie.

**Movie 2** Time‐lapse movie generated of the rate of wound healing/contraction following distal fin amputation under 200 mM oxamate treated condition over 12 min. 360 still images were taken every 2 s over 12 min and rendered as a time‐lapse movie using iMovie at 40 frames per second, giving a 9‐s time‐lapse movie.

**Movie 3** Time‐lapse movie generated of the rate of wound healing/contraction following distal fin amputation under 1% DMSO control treated condition over 12 min. 360 still images were taken every 2 s over 12 min and rendered as a time‐lapse movie using iMovie at 40 frames per second, giving a 9‐s time‐lapse movie.

**Movie 4** Time‐lapse movie generated of the rate of wound healing/contraction following distal fin amputation under 400 μM GNE‐140 treated condition over 12 min. 360 still images were taken every 2 s over 12 min and rendered as a time‐lapse movie using iMovie at 40 frames per second, giving a 9‐s time‐lapse movie.

**Table S1** Laconic/pcDNA3.1(−) was a gift from Luis Felipe Barros (Addgene plasmid #44238; http://n2t.net/addgene:44238; RRID:Addgene_44238) San Martin *et al*.^17^

## Data Availability

Data sharing not applicable to this article as no datasets were generated or analysed during the current study.
