# Supplementary figures and images for: Aerobic glycolysis is important for zebrafish larval wound closure and tail regeneration
Source: Wound Repair Regen. 2022 Oct 5;30(6):665–80. doi: 10.1111/wrr.13050 (PMC9828577; doi:10.1111/wrr.13050)

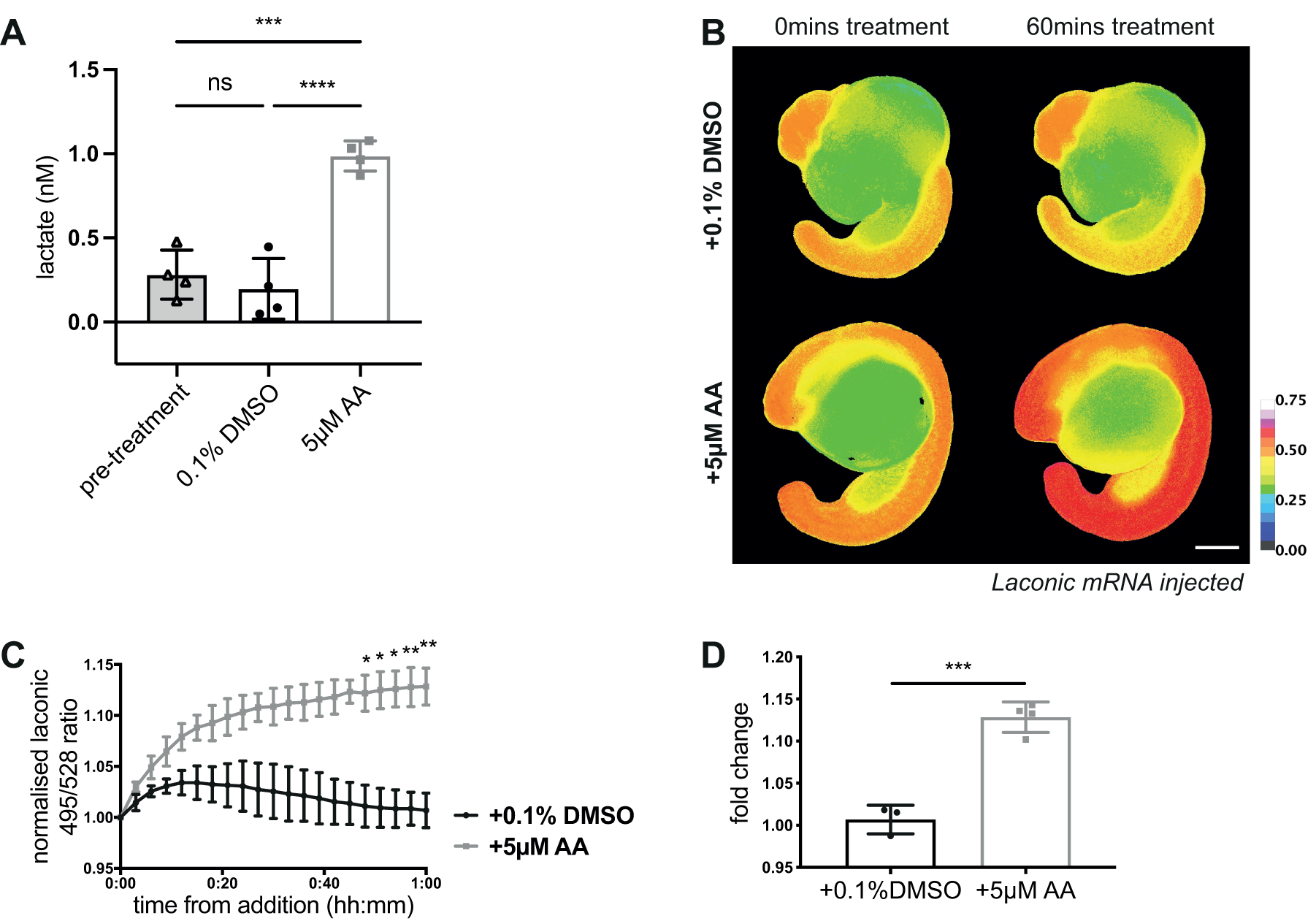

Supplement: Supplementary file 1 — Figure S1 Laconic positive controls as mRNA injections. (A) Graph of lactate levels in 2dpf embryos (calculated using a standard curve) after 10 min of treatment with 0.1% DMSO or 5 μM AA compared to a pre‐treatment baseline level. One‐way ANOVA to calculate significance, n = 36. (B) Micrographs of representative ~19hpf wild‐type embryos injected with laconic mRNA at the one‐cell stage before and after 60 min of treatment with 0.1% DMSO or 5 μM AA, pseudocoloured to show Laconic ratio. (C) Graph showing Laconic ratios over time during treatment with 0.1% DMSO or 5 μM AA, normalised to pre‐treatment value. Two‐way ANOVA to calculate significance, n = 4. (D) Graph showing quantification of ratio change as fold change (post‐treatment ratio divided by pre‐treatment ratio). Students' t‐test to calculate significance, n = 4. All scale bars represent 200 μm. Differences were considered significant to * P < 0.05, ** P < 0.01, *** P < 0.001, **** P < 0.0001, and ns P ≥ 0.05 [file WRR-30-665-s006.tif]

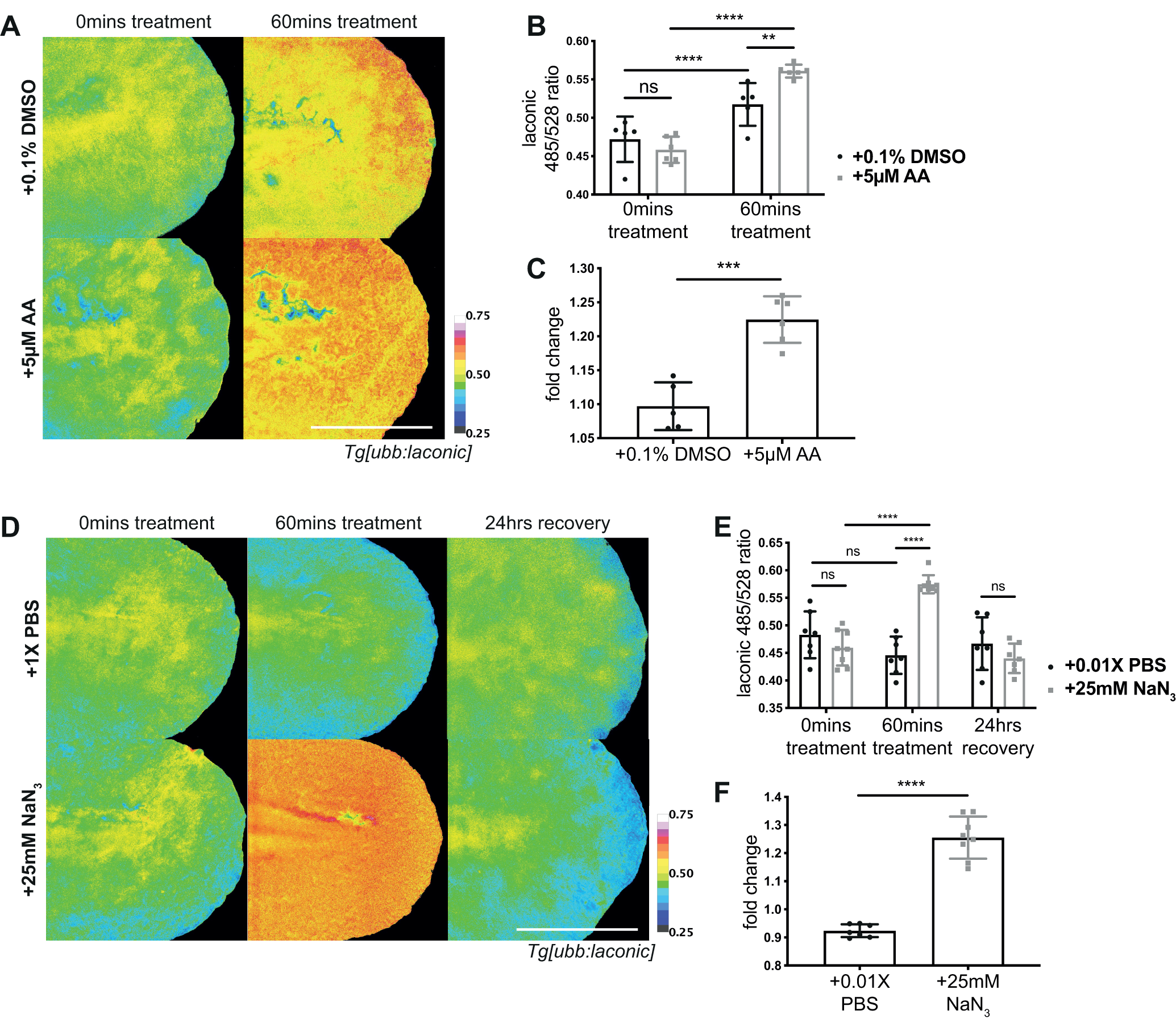

Supplement: Supplementary file 2 — Figure S2 Laconic positive controls as a transgenic line. (A) Micrographs of representative Tg[ubb:laconic] lkc1 embryos tails at 48hpf before and after treatment with 0.1% DMSO or 5 μM AA, pseudocoloured to show Laconic ratio. (B) Graph showing raw Laconic ratios pre‐treatment and after treatment with 0.1% DMSO or 5 μM AA. Two‐way ANOVA to calculate significance, n = 6. (C) Graphs showing quantification of ratio change after 60 min of treatment with 0.1% DMSO or 5 μM AA as fold change (ratio after treatment divided by pre‐treatment value). Students' t‐test to calculate significance, n = 6. (D) Micrographs of representative Tg[ubb:laconic] lkc1 embryos tails at 48hpf before and after 60 min of treatment with 0.01X PBS or 25 mM sodium azide (NaN3), and after 24 h recovery post wash out of the drug, pseudocoloured to show Laconic ratio. (E) Graph showing raw Laconic ratios pre‐treatment, after 60 min of treatment with 0.01X PBS or 25 mM NaN3, and after 24 h of recovery after drug wash out. Two‐way ANOVA was used to calculate significance, n = 8. (F) Graph showing quantification of ratio change after 60 min of treatment with 0.01X PBS or 25 mM NaN3 as fold change (ratio after treatment divided by pre‐treatment value). Students' t‐tests were used to calculate significance, n = 8. All scale bars represent 200 μm. Differences were considered significant to * P < 0.05, ** P < 0.01, *** P < 0.001, **** P < 0.0001, and ns P ≥ 0.05. [file WRR-30-665-s007.tif]

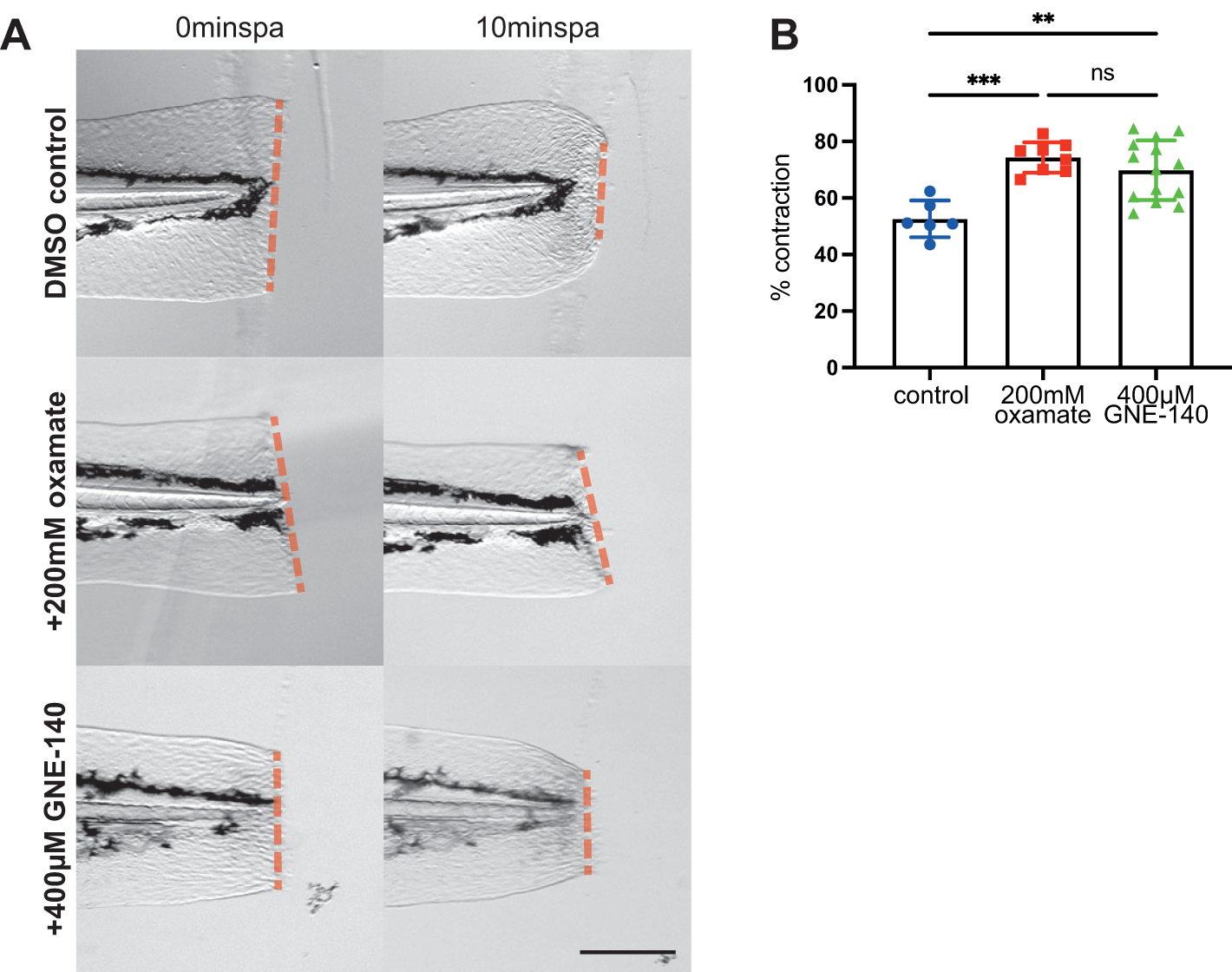

Supplement: Supplementary file 3 — Figure S3 Further inhibition of lactate production during fin fold amputation. (A) Brightfield images of 2dpf WT control (no treatment) compared with 200 mM oxamate‐ and 400 μM GNE‐140‐treated embryos at 0minspa and 20minspa. Red dashed lines indicate example measurements taken for quantification. The scale bar represents 200 μm. (B) Graph showing percentage contraction at 10minspa of wound width at 0minspa. One‐way ANOVA to calculate significance. [file WRR-30-665-s009.tif]

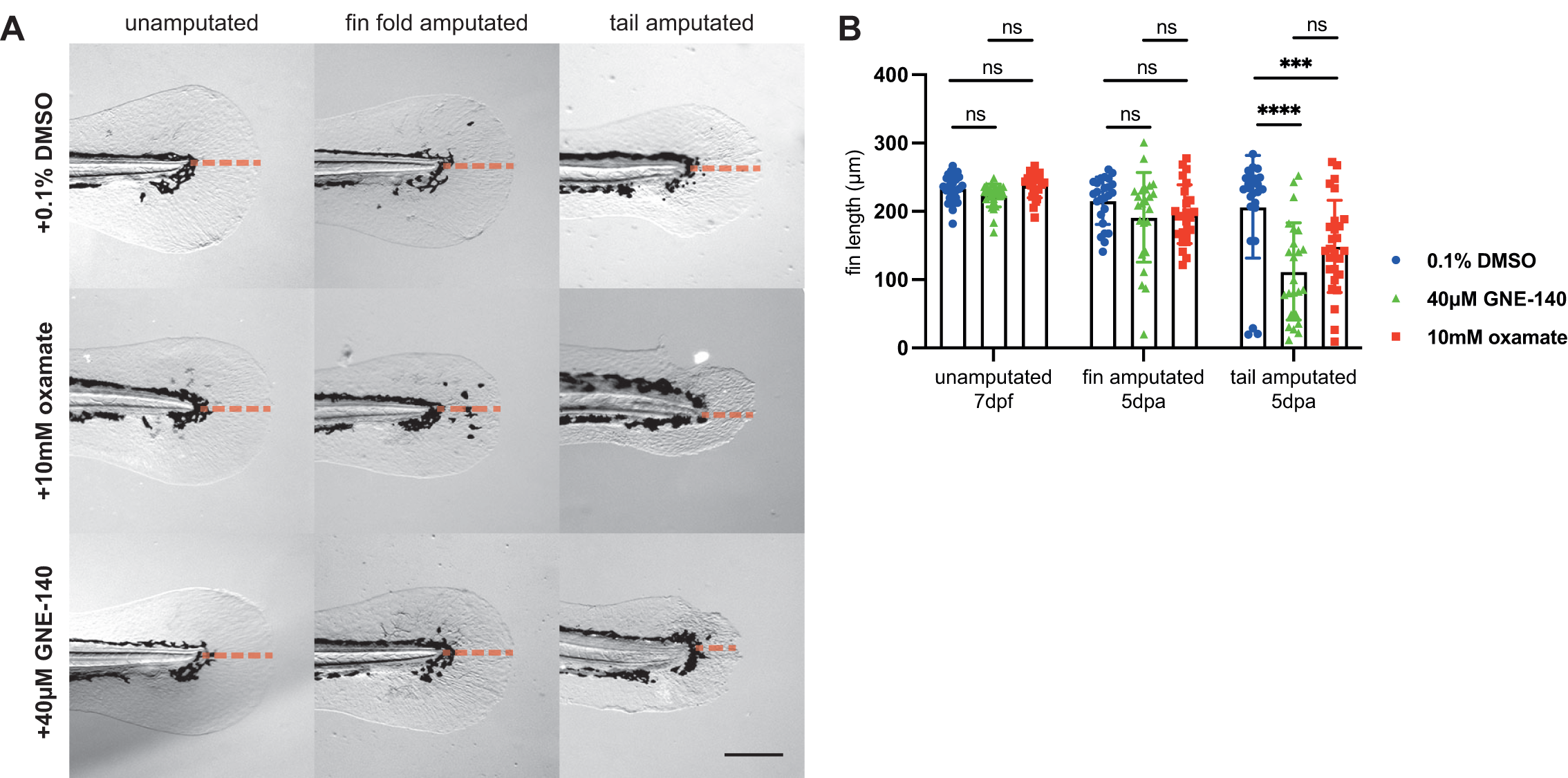

Supplement: Supplementary file 4 — Figure S4 Further inhibition of lactate production over the whole of regeneration. (A) Brightfield images of representative WT embryos at 5dpa (7dpf), treated with 0.1% DMSO (control), 10 mM oxamate, or 40 μM GNE‐140. Red dashed line indicates measurements taken for quantification of regrowth. The scale bar represents 200 μm. (B) Graph comparing inhibited (10 mM oxamate or 40 μM GNE‐140 treatment) with control (0.1% DMSO treatment) embryos in the unamputated, fin fold amputated, and tail amputated conditions at 5dpa (7dpf). Two‐way ANOVA to calculate significance. [file WRR-30-665-s003.tif]
