# Supplementary material for: Aerobic glycolysis is important for zebrafish larval wound closure and tail regeneration
Source: Wound Repair Regen. 2022 Oct 5;30(6):665–80. doi: 10.1111/wrr.13050 (PMC9828577; doi:10.1111/wrr.13050)
Supplement: Supplementary file 9 — Table S1 Laconic/pcDNA3.1(−) was a gift from Luis Felipe Barros (Addgene plasmid #44238; http://n2t.net/addgene:44238; RRID:Addgene_44238) San Martin et al. 17 [file WRR-30-665-s005.docx]

Supplementary Table 1

| Insert | Original backbone | New backbone | Restriction enzyme 5’ | Restriction enzyme 3’ | Double digest conditions | New construct | Notes |
| --- | --- | --- | --- | --- | --- | --- | --- |
| Laconic | pcDNA3.1(-) | pCS2+ | EcoRI | XbaI (site added from HindIII site via short oligos) | Roche buffer H, 37˚C | pCS2-Laconic | 1. Digest pcDNA3.1-Laconic with HindIII (Roche buffer B, 37˚C), XbaI site added by ligation with primers* 2. Digest new pcDNA3.1-Laconic with EcoRI and XbaI (2262bp) 3. Digest pCS2 with EcoRI and XbaI (4074bp) (will create extra 40bp fragment)   * 5’-AGCTGTCTAGACCCA-3’  5’-AGCTTGGGTCTAGAC-3’ |
| Laconic | pcDNA3.1(-) | p3-HyPer | BamHI | HindIII | Roche buffer B, 37˚C | p3-Laconic | 1. Digest Laconic with BamHI and HindIII (2239bp) 2. Digest p3-HyPer with BamHI and HindIII (2967bp) |

Laconic/pcDNA3.1(-) was a gift from Luis Felipe Barros (Addgene plasmid #44238; http://n2t.net/addgene:44238; RRID:Addgene_44238) (San Martin et al., 2013).
